# Development of an oral gut-targeted rabies virus-like particles (RVLPs) vaccine with mucosal immune adjuvant LTB via delivering of localized-release microparticles

**DOI:** 10.1080/22221751.2025.2515406

**Published:** 2025-06-06

**Authors:** Jinping Niu, Zhangting Zhao, Tong Zhang, Qingwei Liu, Liyao Huang, Shipo Li, Haipeng Liu, Shaowen Yu, Linfeng Li, Hao Jia, Wenyun Zheng, Feng Yang, Xingyuan Ma

**Affiliations:** a State Key Laboratory of Bioreactor engineering, East China University of Science and Technology, Shanghai, People’s Republic of China; b Shanghai Key Laboratory of New Drug Design, School of Pharmacy, East China University of Science and Technology, Shanghai, People’s Republic of China; c State Key Laboratory of Marine Environmental Science, Xiamen University, Xiamen, People’s Republic of China; d School of Mathematics, East China University of Science and Technology, Shanghai, People’s Republic of China; e School of Medicine, Shanghai Jiao Tong University, Shanghai, People’s Republic of China; f School of Pharmacy, Naval Medical University, Shanghai, People’s Republic of China

**Keywords:** Oral gut-targeted, rabies virus-like particles (RVLPs), mucosal immune vaccines, localized-release microparticles, LTB B subunit of heat-labile toxin (LTB), PLGA/Eudradit microparticle (EPLGA MPs)

## Abstract

Rabies, a fatal zoonotic infectious diseases caused by rabies virus (RABV) infection, still has a high incidence with no effective cure in many Asian countries, even though numerous commercial vaccines have been administered for decades. One of the most important reasons is the neglected that main reservoirs of RABV, such as many stray and wild animals, are inaccessible for effective vaccination, especially in natural wilderness environments. In this study, we developed a highly effective gut-targeted oral rabies vaccine (ORV), which containing the immunoadjuvant LTB by targeted administration of microparticles with local release in the intestine. Based on the virus like particles (RVLPs) were assembled by RABV glycoprotein (RVGP) and matrix protein (RVMP), the enterically released microparticles ELPGA MPs loaded with RVLPs and djuvant LTB (RVLPs + LTB/EPLGA MPs) were prepared and demonstrated the ability of intestinal targeting which released in a pH-dependent manner. Subsequently, *in vivo* immunoassay experiments showed that the levels of anti-RVLPs IgG, IFN-γ and IL-4 were significantly higher in the RVLPs + LTB/EPLGA MPs groups than in the normal saline group or positive control group (R group) after intragastric administration. Moreover, higher levels of CD4+/CD8+ T cells ratios in the peripheral blood and sIgA in the intestines and feces of mice indicated that RVLPs + LTB/EPLGA MPs group elicited a stronger cellular immune response and mucosal immunity. In short, the novel oral vaccine is exploring valuable strategies of oral gut-targeted vaccines and promising to effectively prevent the spread of RABV among terrestrial carnivorous animals and human populations.

## GRAPHICAL ABSTRACT

**Figure:**
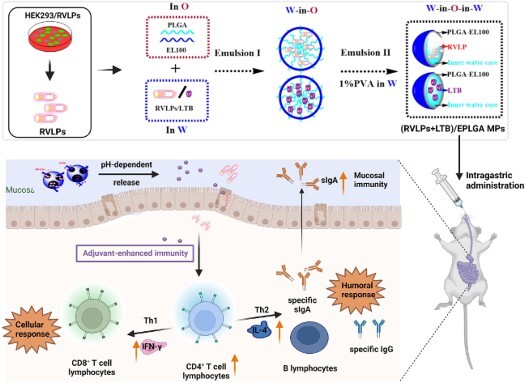

A schematic illustrating the obtain of antigen and its oral delivery mechanism. The RVLPs + LTB/EPLGA MPs were administered intragastric to achieve intestinal targeting and pH-responsive release and induce antigen-specific mucosal, humoral, and cellular immune responses on the intestinal mucosal surface.

## Introduction

To date, rabies remains one of the lethal zoonotic diseases caused by the rabies virus (RABV) with a global distribution. Historical records of rabies can be traced back to at least 4000 years ago [1]. There are many reservoirs of RABV [2,3], however, more than 99% of human rabies cases are attributed to the stray dogs [4]. Fortunately, rabies can be completely averted by the vaccination [3]. In 2018, the World Health Organization (WHO) and its collaborators jointly proposed a global strategic plan to eliminate human rabies transmitted from dogs by 2030, suggesting that mass vaccination of dogs is a promising tool [5]. In developed countries, mass parenteral vaccination of dogs combined with strict preventive measures has shown to be effective in eliminating canine rabies [2]. However, the parenteral rabies vaccine has several limitations. Firstly, it is expensive. Secondly, it needs to be distributed and stored at low temperatures. Thirdly, it is mainly administered to domestic animals by injection, which is inappropriate for stray and wild animals, as they have a wide and undetermined range of activities, making it very difficult to gather them together for a large-scale vaccination campaign, and follow-up monitoring of vaccination is impossible. Therefore, the vaccine coverage is low and less effective in many developing countries with inadequate infrastructure. Rabies-related deaths still account for nearly 60,000 fatalities worldwide each year, a figure which is probably an underestimate [3,6,7].

The emergence of oral wildlife rabies vaccines (ORVs) has opened a new chapter in addressing the shortcomings of existing vaccines. Two oral bait products are available for the control of wildlife rabies, namely RABORAL-RG® (vaccinia-rabies recombinant oral glycoprotein vaccine in a fishmeal-coated sachet) and ONRAB® (adenovirus-rabies recombinant oral glycoprotein vaccine in an ultralight bait matrix) [8,9]. In Europe and North America, the use of ORV to vaccinate wildlife has been demonstrated an inexpensive but effective approach to eradicating or managing wildlife rabies [8]. However, current ORV has never been utilized to prevent dog rabies, despite being the mainstay of eliminating rabies virus from wildlife due to containing live virulent ingredients that can infect human contacts [10].

The development of next-generation ORVs should focus on the safer and more cost-effective subunit vaccines. Traditional live and attenuated vaccines carry risks such as inflammation, reversion and infection, whereas inactivated vaccines usually require additional doses to boost immunity [11]. Virus-like particles (VLPs), a type of subunit vaccine with a supramolecular protein, can better resist the adverse environment encountered during oral or nasal administration, making it an attractive target for this type of immunity [12]. In addition, VLPs vaccines combine many advantages of whole virus and recombinant subunit protein vaccines [13], such as mimicking the way the virus stimulates the immune response without the risk of replication or spread [14,15], displaying highly repetitive epitopes on the surface to promote B cell receptor (BCR) cross-linking and induce a robust B cell response [13,16], and enhancing and modulating the immune response by being a potential adjuvant [16,17]. Currently, several VLP vaccines have been approved by the Food and Drug Administration (FDA) for human use, including hepatitis B virus and human papillomavirus [16]. However, while there exist several investigations regarding rabies VLPs (RVLPs) vaccines [18–21], there are no reports to date on oral rabies virus-like particles (RVLPs) vaccines.

RVLPs are self-assembled from rabies virus glycoprotein (RVGP) and matrix protein (RVMP). Moreover, RVGP is displayed as a major antigen on the surface of RVLPs. To successfully deliver the oral vaccine by oral administration, delivery vehicles that co-deliver sustained-release antigens and mucosal immune adjuvants are becoming research hotspots [11]. Mucosal adjuvants can significantly enhance the immune effect of antigen, including cytokines, granulocyte-macrophage colony-stimulating factor (GM-CSF), CpG-ODN, and bacterial toxins [22,23]. Among these, heat-labile toxin (LT) is one of the most promising toxin-mediated adjuvants so far [24]. To avoid its endogenous toxicity, the enhancement effect of B subunit of LT (LTB) isolated from LT on the immune response to co-administered antigen has been explored [25]. To enable the packaged antigen and adjuvant to be orally delivered to the intestine and released in a controlled manner, we can coat Eudragit®RLPO18, Eudragit®L10020, or Eudragit®FS30D21 on the surface of PLGA nanoparticles (NPs). PLGA has been extensively employed in vaccine delivery due to its good histocompatibility and biodegradability, which can enable sustained release of antigens in a neutral environment [26,27]. Nevertheless, PLGA NPs has been found to be unstable in the acidifying microenvironment, leading to the destruction and degradation of the tertiary structure of the encapsulated protein [28]. Although Eudragit is an acid-resistant, enteric-coated, sustained-release material, it can be used combined with PLGA to overcome the shortcoming of PLGA NPs’ instability in acidic environments [29].

To develop a novel RVLPs vaccine for eliciting strong immune responses by oral inoculation for terrestrial carnivorous animals as the main infectious source of rabies, especially those stray animals and wild animals that have not yet been vaccinated, this research was designed to engineer an oral gut-targeted virus-like particles (VLPs) rabies vaccine via delivery of localized-release microparticles. Guided by mucosal immunological theory, we will utilize an advanced oral gut-targeted vaccine delivery system, an efficient mucosal immune adjuvant (LTB) and optimized self-assembed antigens to enable it to exert optimal immune effects. Through this study, it is expected not only to prevent the spread of rabies virus among various animals to reduce the risk of zoonosis to humans, but also to lay the foundation for developing other new oral VLP subunit vaccines, such as oral COVID-19 vaccines, which could help to end this pandemic faster.

## Results

### Rational analysis of RVGP from CVS strain

As the only effective antigen present on the surface of RABV, mature RVGP consists of 505 aa and is divided into an extramembrane domain (20–460aa), a transmembrane domain (460–480aa), and a cytoplasmic tail (480–524aa) [30]. Interestingly, the extracellular domain of RVGP contained all antigenic epitopes and three potential N-glycosylation sites (Asn56, Asn266, and Asn338) (Figure S1(a)). Glycosylation is very important for the tertiary structure and immunogenicity of the antigen protein [31]. Furthermore, Asn56 was included in specific B cell epitopes, whereas Asn266 and Asn338 were contained in linear B and CTL cell epitopes, indicating that the extracellular domain plays a key role in antigenicity (Figure S1(a)). When Asn56 undergoes mutated, the specific B-cell epitope is lost, and the secondary structure undergoes a transformation from *β*-sheets to coils (Figure S1(c,d)). In contrast, the mutations of Asn266 and Asn338 have little effect on the cell epitope and tertiary structure of RVGP (Figure S1(c,d)). The above results confirmed that N-glycosylation affects the tertiary structure and antigenicity of RVGP. Therefore, a suitable expression system with correct post-translational modification is crucial to produce RVGP. Additionally, there is 97.25% sequence consistency between RVGP from the CVS strain and another 34 RVGPs from different regions of China (Figure S1(b)), indicating that the RVGP from the CVS strain could be used as a broad-spectrum antigen against Chinese animal rabies and further applied around the world.

### Screening and identification of positive monoclonal cell line HEK-293/RVLPs

VLPs with highly repetitive epitopes are more stable and immunogenic than simple recombinant proteins [32]. Since RVGP and RVMP can self-assemble into RVLPs, they were constructed in the pcDNA3.1(+) vector together with the EGFP protein (Graphic Abstract). Besides, EGFP was also used as an auxiliary tool to identify and characterize stable cell lines. To enhance the mucosal immune effect of RVLPs, the mucosal adjuvant LTB was expressed. LTB was mainly expressed in the form of inclusion bodies, and 89.15% of LTB monomers could be assembled into active pentamers after renaturation (Figure S2(a–c)).

**Figure 1.**
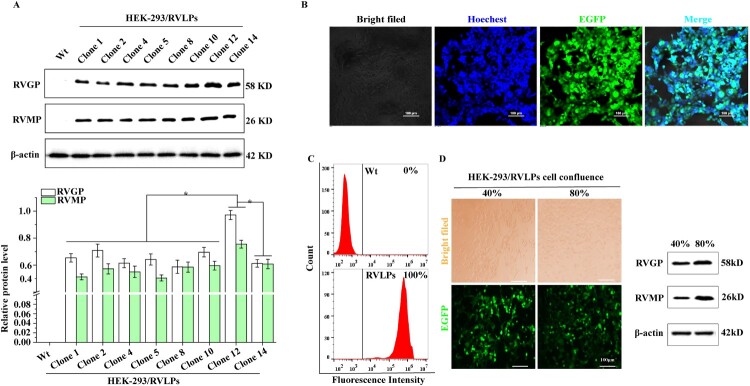
Identification of HEK-293/RVLPs positive monoclonal cell lines. (a) Protein levels of RVLPs in different positive monoclonal cell lines. The stability of HEK-293/RVLPs cell line identified by CLSM (b) and FCM (c). (d) The expression level of RVLPs in different confluence of HEK-293/RVLPs cell lines. All the images were taken at 100× magnification (Bar = 100 µm). Data were expressed as mean ± SD (*n* = 3). *P < 0.05.

The transfection efficiency of the pcDNA3.1(+)-RVLPs-EGFP plasmid reached 65.66% (Figure S3(a)). Furthermore, the expression of RVLPs could be detected by qPCR and WB in HEK-293 cells after transfection (Figure S3(b)). Interestingly, the protein of RVGP and RVMP could be detected in both HEK-293 cells and their culture medium (Figure S3(d)), confirming that RVLPs can be secreted into the medium after assembly. Subsequently, G418 was used to screen stable cell lines (Figure S3(c,d)). After 14 days of continuous screening, the positive HEK-293/RVLPs monoclonal cell was then obtained and expanded by means of a limiting dilution method. The cell lines with a single peak of EGFP were screened by flow cytometry and named HEK-293/RVLPs clone 1/2/4/5/8/10/11/12/14 (Figure S3(e)). Furthermore, the average fluorescence intensity and protein expression level of clone 12 were significantly higher than other positive monoclonal cell lines (*P* < 0.05) (Figure S3(e) and Figure 1(a)). Therefore, clone 12 was used in subsequent research and named HEK-293/RVLPs.

To evaluate the stability of the HEK-293/RVLPs cell line, it was digested for confocal laser scanning microscope (CLSM), flow cytometry (FCM), and western blot assays at P10 generation. The results showed that the proportion of EGFP-positive cells reached 100% (Figure 1(b,c)). Furthermore, western blot analysis of HEK-293/RVLPs cells showed consistent intracellular expression levels of RVLPs (Figure 1(d)). Collectively, these findings validate the robust stability of HEK-293/RVLPs cells as an efficient RVLPs expression system.

### Preparation and characterization of RVLPs/EPLGA-MPs

**Figure 2.**
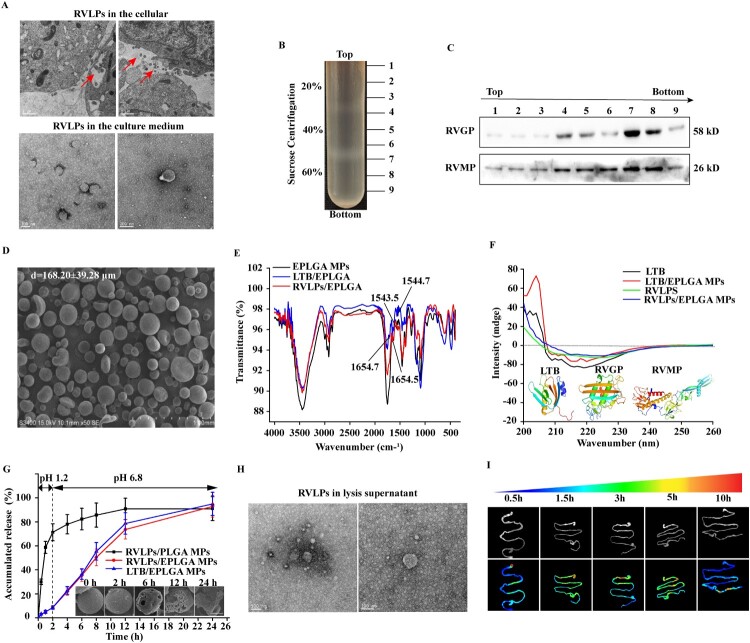
Preparation and characterization of RVLPs/EPLGA MPs. (a) The morphology of RVLPs observed by TEM. (b) Purification of RVLPs by continuous sucrose density gradient centrifugation. (c) The continuous sucrose density gradient samples were analyzed by western blot. (d) The morphology of RVLPs/EPLGA MPs. (e) FTIR spectra of RVLPs/EPLGA MPs. (f) CD spectra of RVLPs/EPLGA MPs. (g) The release profile of RVLPs from EPLGA MPs. (h) The morphology of RVLPs in the lysate supernatant. (i) *In vivo* image of gastrointestinal tract of mice after oral administration of mCherry/EPLGA MPs. Red represents the location of mCherry/EPLGA MPs in the gastrointestinal tract of mice.

RVLPs, with a size range of 180–200 nm, were observed within the vesicles and outside of HEK-293/RVLPs cells and their culture medium (Figure 2(a)). Following purification, RVLPs were detected in all sucrose layers (Figure 2(c)), indicating varying sedimentation coefficients. Notably, the majority of RVLPs were found in the 40–60% sucrose layer (Figure 2(b)), consistent with the sedimentation coefficient of natural RABV. Therefore, only RVLPs located between the 40–60% sucrose layers were collected.

RVLP/EPLGA MPs were prepared with a smooth surface and a relatively regular spherical morphology after being vacuum freeze-drying (Figure 2(d)). Additionally, the average particle size and encapsulation efficiency (EE) of RVLPs/EPLGA MPs were ranged from 168.20 ± 39.28 µm to 97.14 ± 1.37%, respectively (Figure 2(d)). To further demonstrate that RVLPs/LTB were encapsulated within EPLGA MPs, the amide bonds in the protein were detected by FTIR. Compared with EPLGA MPs, LTB/EPLGA MPs had a polarization peak at 1544.7 and 1654.7 cm^−1^, whereas the polarization peaks in RVLPs/EPLGA MPs were found at 1543.5 and 1654.5 cm^−1^ (Figure 2(e)). The two polarization peaks are generated by bending vibrations of the protein amide I bond (C = O) and II bond (N–H), respectively [33]. Hence, LTB and RVLPs were successfully encapsulated in EPLGA MPs. Furthermore, the secondary structure of a protein can be effectively evaluated for intact integrity by using CD spectra [34]. As shown in Figure 2(f), both far-UV and near-UV spectra of RVLPs/LTB released from EPLGA MPs displayed similar secondary structures to the native ones (Figure 2(f)), indicating that LTB and RVLPs could be successfully encapsulated in EPLGA MPs without destroying their structure. In addition, RVLPs/EPLGA MPs could be stored at 4°C for at least 3 months (Figure S4).

The release profile of RVLP/EPLGA MPs was evaluated in the stimulated conditions of the stomach and intestine. The EPLGA MPs exhibited a pH-responsive characteristic in contrast to PLGA MPs, merely less than 10% of RVLPs/LTB were released from EPLGA MPs within 2 h at pH 1.2, while nearly 70% of RVLPs were released from PLGA MPs. Although the cumulative release rate (nearly 95%) was similar between these two types of MPs at 24 h, EPLGA MPs effectively achieved the goal of slowing the release of most of the target protein at pH 6.8 environment (Figure 2(g)). Therefore, EPLGA MPs could serve as a carrier that protects loaded antigens from the harsh environment of the stomach by limiting the release of antigens in the gastric fluid, while slowly releasing antigens in the intestine. Importantly, the morphology of released RVLPs corresponded with the native ones (Figure 2(h)), fully demonstrating the stability of antigen in EPLGA MPs.

To verify the intestinal targeting ability of acid-resistant EPLGA MPs. After intragastric administration of mCherry/EPLGA MPs, the gastrointestinal tracts of mice were harvested at a specific time. As shown in Figure 2(i), mCherry/EPLGA MPs were mainly retained in the stomach at 0.5 h, followed by gradual transit to the duodenum at 1.5 h and complete arrival in the small intestine at 3 h. Subsequently, mCherry/EPLGA MPs gradually left from the duodenum to the ileum. After 10 h of oral administration, mCherry/EPLGA MPs reached the colon. These results further demonstrated that encapsulated proteins could avoid the harsh environment of the stomach and target the intestine.

### Humoral immunity and cellular immunity induced by RVLPs + LTB/EPLGA MPs in mice

**Figure 3.**
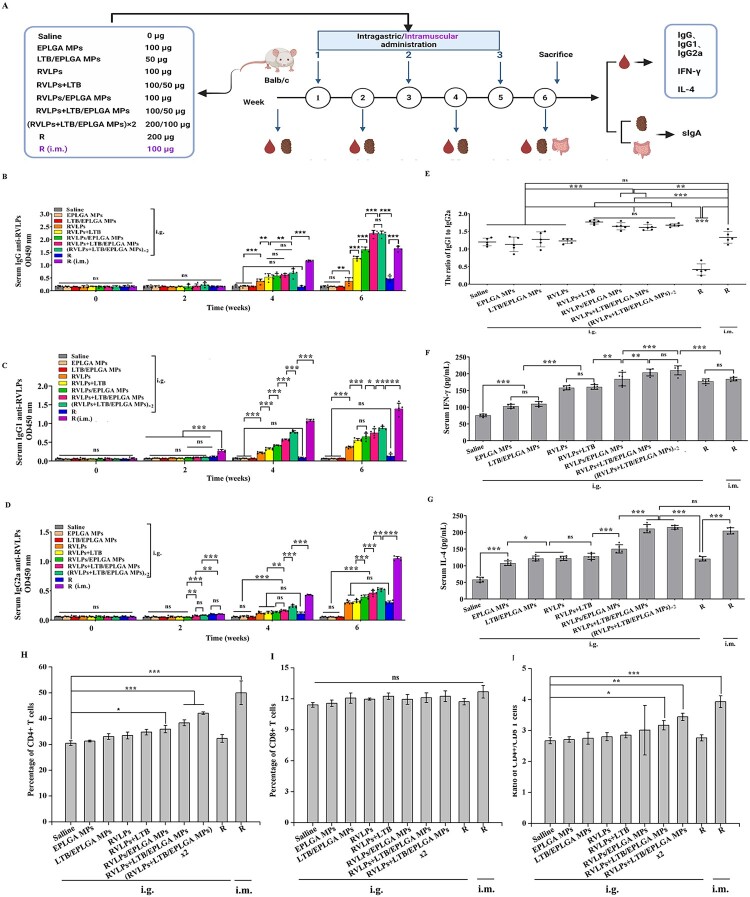
RVLP + LTB/EPLGA MPs induced potent humoral immunity and cell immunity. (a) Schematic diagram of animal immunity. The levels of anti-RVLPs antibodies (b) IgG, (c) IgG1, and (d) IgG2a. (e) The ratio of IgG1 to IgG2a. (f) The concentrations of IFN-γ in the serum of immunized mice. (g) The concentrations of IL-4 in immunized mice serum. (h) The proportion of CD4+ T cells in the peripheral blood of immunized mice. Neutralizing antibody titers against RVLPs in the serum of immunized mice. (i) The proportion of CD8+ T cells in the peripheral blood of immunized mice. (j) The CD4+/CD8+ T cells ratio in the peripheral blood of immunized mice. Data were expressed as mean ± SD (*n* = 3). *P < 0.05, **P < 0.01, and ***P < 0.001, and ns represented not significant. *R* represented Rabisin.

To evaluate the immunogenicity of RVLPs + LTB/EPLGA MPs, Balb/c mice (*n* = 5/group) were orally immunized, and RVLPs-specific antibody levels as well as inflammatory factors in serum were quantified using ELISA (Figure 3(a)). The presence of serum-specific antibodies IgG and its subtypes may partially indicate the extent of humoral immune response. As shown in Figure 3(b), the level of anti-RVLPs IgG antibodies increased with each subsequent immunization in the experimental groups. After the third immunization, both RVLPs + LTB/EPLGA MPs and (RVLPs + LTB/EPLGA MPs) ×2 groups, induced the highest anti-RVLPs IgG antibody response with no statistical difference between them, which was 1.39 times that of RVLPs/EPLGA MPs (*P* < 0.001). The above results indicated that oral RVLPs + LTB/EPLGA MPs could induce RVLPs-specific humoral immune response, and the adjuvant LTB could significantly enhance the immune response.

To assess the roles of both subsets of T helper cells in the immune response, the level of antigen-specific IgG1 and IgG2a antibody in serum was analyzed (Figure 3(c,d)). All immunized mice in RVLPs/EPLGA MPs, RVLPs + LTB/EPLGA MPs and (RVLPs + LTB/EPLGA MPs) ×2 groups exhibited elevated IgG1/IgG2a ratios, indicating a Th2-based immune response. Furthermore, it has been reported that Th1 effector cells can produce interferon-γ (IFN-γ) and mediate cellular responses against intracellular pathogens. Th2 effector cells produce IL-4, which regulates humoral immune responses [35]. The concentration changes of IFN-γ and IL-4 in the serum were similar (Figure 3(f,g)). Compared with the saline group, the levels of IFN-γ and IL-4 were highest in the RVLPs + LTB/EPLGA MPs and (RVLPs + LTB/EPLGA MPs) ×2 groups (*P* < 0.001). Moreover, the INF-γ and IL-4 levels of RVLPs + LTB/EPLGA MPs group were 1.14 (*P* < 0.01) and 1.43 times (*P* < 0.001) higher than those of RVLPs/EPLGA MPs group. The above results indicated that LTB could enhance the expression level of serum cytokines. Subsequentely, similar results were found in the detection the levels of CD4+ T and CD8+ T in the peripheral blood of the mice (Figure 3(h,i)), indicating that RVLPs + LTB/EPLGA MPs could induce a significant cellular immune response. Studies have shown that when the ratio of CD4+/CD8+ T cells increases, it indicates that the immune response of memory T lymphocytes is activated [36]. In this study, the ratio of CD4+ to CD8+ T cells in RVLPs + LTB/EPLGA MPs and (RVLPs + LTB/EPLGA MPs) ×2 increased significantly (Figure 3(j)), indicating the immune response of memory T lymphocytes is activated by RVLPs + LTB/EPLGA MPs.

### Mucosal immunity induced by RVLPs + LTB/EPLGA MPs in mice

**Figure 4.**
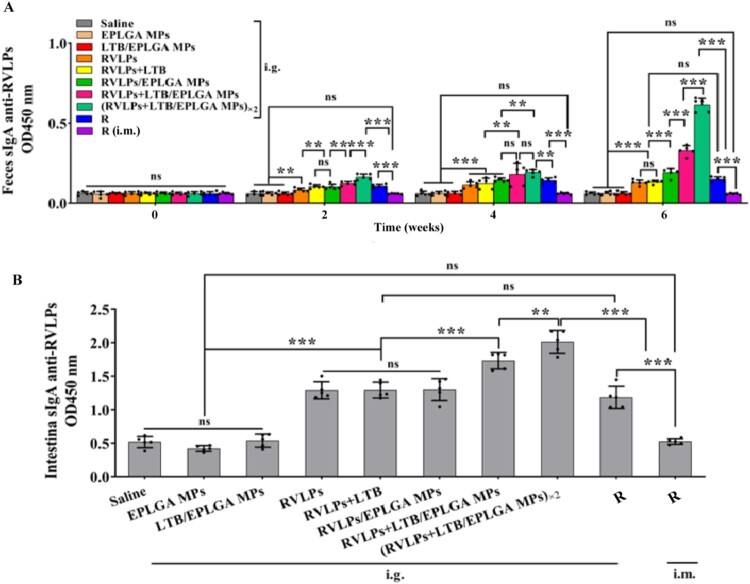
RVLP + LTB/EPLGA MPs induced robust potent mucosal immunity. The anti-RVLPs specific sIgA antibody in (a) feces and (b) intestinal. Data were expressed as mean ± SD (*n* = 3). *P < 0.05, **P < 0.01, ***P < 0.001, and ns represented not significant.

**Figure 5.**
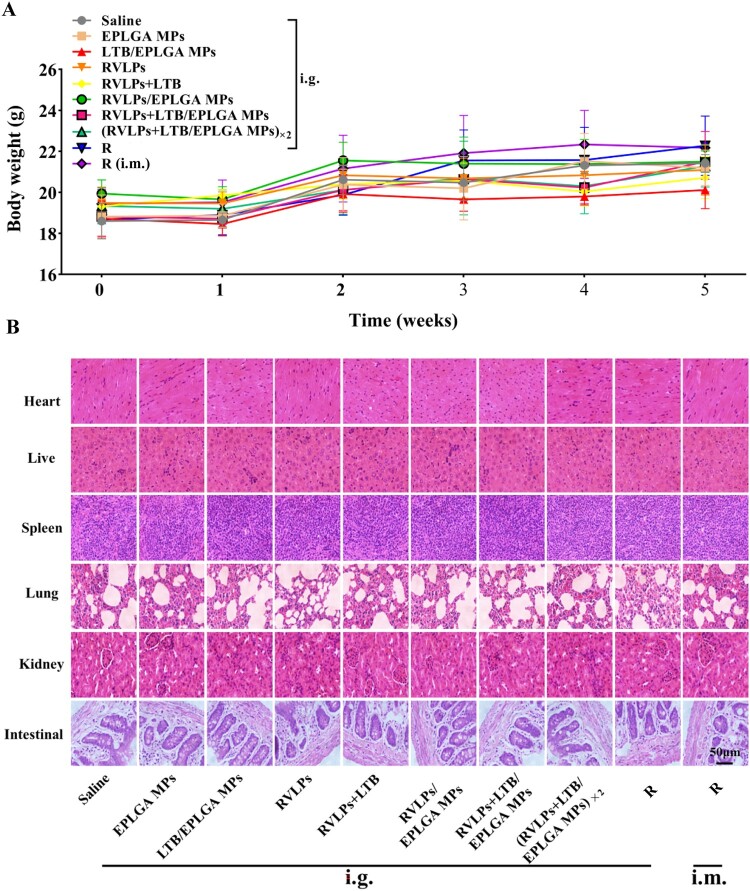
Biosafety evaluation of mice in different administration groups. (a) The changes of body weight. (b) Histological examination of major organs of mice. All the images were taken at 400× magnification (Bar = 50 µm)

The level of mucosal immune response elicited by the oral vaccine can be assessed by analyzing the level of RVLPs-specific sIgA antibodies in the feces and intestinal fluid of the mice. As shown in Figure 4, the EPLGA MPs and LTB/EPLGA MPs groups are unable to induce mucosal-specific sIgA antibodies. The contents of RVLPs-specific sIgA antibodies in the feces of all experimental groups increased significantly with the number of immunizations, with the (RVLPs + LTB/EPLGA MPs) ×2 group showing the most significant increase (Figure 4(a)). After the primary immunization, the sIgA level of the (RVLPs + LTB/EPLGA MPs) ×2 group was 1.33 times (*P* < 0.001) higher than that of the RVLPs + LTB/EPLGA MPs group. After the second immunization, there was no statistical difference between the sIgA levels of the RVLPs + LTB/EPLGA MPs and (RVLPs + LTB/EPLGA MPs) ×2 group, but the (RVLPs + LTB/EPLGA MPs) ×2 group was 1.32 times (*P* < 0.01) higher than the RVLPs/EPLGA MPs group. After the third immunization, the level of sIgA in the (RVLPs + LTB/EPLGA MPs) ×2 group was 1.87 times (*P* < 0.001) higher than that in the RVLPs + LTB/EPLGA MPs group. Moreover, after the third immunization, the levels of specific sIgA antibodies in the intestinal fluid of all experimental groups were significantly increased (*P* < 0.001). Among them, the (RVLPs + LTB/EPLGA MPs) ×2 group elicited the most significant specific mucosal immune response, which was 1.16-fold (*P* < 0.01) higher than that of the RVLPs + LTB/EPLGA MPs group. Excitingly, mucosal immunity induced by RVLPs + LTB/EPLGA MPs was stronger than that induced by injection or oral Rabisin (Figure 4(b)). Moreover, the weight of the mice and the HE staining results of the main organs showed that the vaccine components were safe (Figure 5).

The above results confirmed that the oral RVLPs could stimulate the production of the mucosal sIgA antibodies, while the LTB adjuvant could enhance the level of mucosal immune response. The best mucosal immune effect was achieved when (RVLPs + LTB/EPLGA MPs) ×2 was administered orally. Due to the sustained release properties of EPLGA MPs, continuous stimulation of antigens could be achieved, resulting in a longer-lasting immune response.

In summary, a stable HEK-293 cell line overexpressing RVLPs was screened and identified based on the selection of candidate RVLPs targeting more than 97% of the major RABV animals in China. When RVLPs were encapsulated by EPLGA MPs and administrated orally, they could induce a stronger antigen-specific immune response. When RVLPs/EPLGA MPs were co-administered with LTB/EPLGA MPs, the immune response was enhanced. Except for specific humoral immunity, the RVLPs-specific cellular and mucosal immune responses increased with the increasing of the dose of antigen and adjuvant. Besides, the level of humoral, cellular, and mucosal immune response produced by (RVLPs + LTB/EPLGA MPs) ×2 was the highest, indicating that (RVLPs + LTB/EPLGA MPs) ×2 could be used as a potential oral vaccine to prevent the spread of RABV from animals, thus the safety of human beings is improved.

## Discussion

Oral vaccines are an effective means of expanding immunization coverage to control and eliminate RABV that is still a serious disease in Asia. RABV is a highly potent neurotropic negative-strand RNA virus that can cause acute encephalomyelitis in all warm-blooded animals including humans [37]. Once RABV retrogrades to the central nervous system (CNS), the mortality rate is almost 100% [38]. Nearly 60,000 people die of rabies each year worldwide, including more than 3000 people in China. In China, 84% of rabies-related deaths occur in rural areas, and stray dogs are the main reservoir response for more than 99% of human cases [39]. Vaccination of stray dogs and other animals is a promising strategy for prevent rabies. The immunization coverage of more than 70% may be sufficient to prevent the transmission of rabies to humans [40]. Oral administration is a desirable way to achieve massive immunization. Although current ORV exhibits a good immunogenicity in most wildlife, its low cross-protection severely limits its widespread application. Besides, ORV has not been used to eliminate dog rabies, because there are many publications and field reports highlighting the deficiencies of using ORV in susceptible dog subgroups [2].

Oligosaccharide chains were necessary for the correct folding and immunogenicity of RVGP, which is an optimal antigen for the development of ORV, as it is the only glycosylated antigen capable of inducing neutralizing antibodies [41]. However, the majority of the antigenic sites of RVGP are conformational epitopes that depend on the correct quaternary protein structure [41]. Our results showed that oligosaccharide chains are necessary for the correct folding and immunogenicity of RVGP. Upon mutating the N-glycosylation site of RVGP, the spatial structure of RVGP changed and the spatial epitope was lost (Figure S1(c,d)). This might be attributed to the fact that the oligosaccharide chains increasing the solubility of folding intermediates during the synthesis of the endoplasmic reticulum and causing RVGP to interact with cadherin and calreticulin. Cadherin and calreticulin have lectin properties and promote the correct folding of RVGP. Moreover, proper glycosylation can improve the characteristics of recombinant proteins, such as increasing their stability and circulation half-life [42,43]. Furthermore, RVGP can self-assemble with RVMP to form RVLPs [7]. RVLPs, with a high density of cell epitopes, have an intrinsic size (200 × 75 nm) (Figure 2(a)) that is suitable for presentation by M cells in the gut and elicits robust cellular and humoral responses as a direct immunogen [44].

Novel PLGA/Eudragit microparticles played a key role in the delivery of RVLPs for gut-targeted release. Although numerous studies have been devoted to the development of the VLPs vaccine containing RVGP, it is injectable and thus not suitable for large-scale vaccination, while oral vaccination is suitable [45,46]. In this study, RVLPs were administered by the oral route. However, naked RVLPs can undergo denaturation and degradation in the stomach due to the acidic pH and enzymes, reducing the effecacy of antigens to stimulate specific immune responses. Polymeric delivery systems have been widely used to protect subunit vaccines. Chitosan is a natural cationic polymer characterized by adhesiveness, biodegradability and compatibility. Its amino and carboxyl groups can form hydrogen bonds with glycoproteins in the mucus layer to make it adhere to the intestinal mucosa, so it has been used to deliver protein drugs to improve their bioavailability and therapeutic effect [47]. Importantly, chitosan directly interacts with intestinal epithelial cells, leading to a decrease in transepithelial resistance and an increase in paracellular permeability [11]. The liposome is another type of vaccine delivery vehicle. It has a lipid bilayer structure similar to cell membranes, which prevents the degradation of protein drugs, prolongs the residence time in the body and improves the cell uptake rate [48]. However, liposomal formulations are prone to degradation and instability, which can lead to the sudden release of encapsulated drugs [49]. PLGA is approved by the FDA for human use [50]. The stability of PLGA in the gastrointestinal tract is better than that of liposomes, and PEG-modified PLGA reduces the sudden release effect to some extent [30]. However, none of the above three materials can remain stable under low pH conditions, which may compromise the integrity of protein antigens [51]. Previous research has demonstrated that by coating PLGA NPs with Eudragit®FS30DP, antigens can be selectively delivered to the colon. Compared to PLGA NPs, PLGA/Eudragit NPs formulations elicit immune responses not only in the rectum but also in the vaginal mucosa, ultimately protecting the rectum or vagina from viral attack [52].

In this study, a novel PLGA/Eudradit microparticle system (EPLGA MPs) was prepared as a delivery system. Different from the above study, Eudragit® L100, composed of polymethacrylic acid and ethyl acrylate in a 1:1 ratio, is an anionic polymer that dissolves in intestinal fluid at pH > 6.5. RVLP/EPLGA MPs were prepared with a smooth surface and uniform size (168.20 ± 39.28 µm) (Figure 2(d)). Furthermore, the RVLPs encapsulated within EPLGA MPs had a native tertiary structure and could be stably stored at 4°C for at least 3 months (Figure 2(e,f) and S4). The intestine is the largest mucosal tissue in the body. *In vivo* experiments demonstrated that EPLGA MPs could successfully target the intestinal tract, where they would gradually dissolve and release RVLPs under intestinal pH conditions. The cumulative release of RVLPs in the intestinal tract was approximately 85% within 22 h (Figure 2(g–i)).

Mucosal adjuvant LTB co-administered with antigen stimulated powerful mucosal-specific immune responses in the gut and induced the strong humoral and cellular responses. An adjuvant is one of the most crucial factors in improving the efficacy of oral immunization. LTB is a potent mucosal and parenteral adjuvant that can enhance the immune response to antigens [53]. Due to the high affinity of LTB with gangliosides that are present on the surface of all mammalian cells, the co-administered antigen can be rapidly recognized and presented by APC [25]. Previous studies showed that LTB can enhance RABV-specific humoral and cellular immune responses in mice and dogs by intramuscular administration [20]. In this study, EPLGA MPs were prepared to be encapsulate with both RVLPs and LTB. After oral administration, they could induce not only antigen-specific humoral and cellular immune responses, but also mucosal-specific immune responses. (RVLPs + LTB/EPLGA MPs) ×2 induced the best humoral, cellular, and mucosal immune responses compared with the other experimental groups (*P* < 0.001). CD4+ T cells can be activated and differentiated into Th1 and Th2 cell types. Th1 cells can secrete IFN-γ and modulate cellular responses, and Th2 cells can secrete IL-4 and regulate the humoral immune responses. The RVLPs + LTB/EPLGA MPs group induced the highest levels of IFN-γ and IL-4 (*P* < 0.001) and tended towards the Th2 cell type. What's more, the body weight of the mice and the HE staining results of the major organs showed that the vaccine components had good safety. The above results indicated that RVLPs + LTB/EPLGA MPs could be used as a candidate antigen for oral immunization of domestic animals. Additionally, this study can provide a new research direction for achieving the of large-scale oral immunization in animals.

## Conclusion

In summary, EPLGA MPs were prepared with high encapsulation efficiency of RVLPs (97.14 ± 1.37%) and provided protection against the harsh gastrointestinal environment. Moreover, the EPLGA MPs could target the intestine and release the encapsulated RVLPs gradually within a pH-responsive manner in simulated intestinal fluid. The conformational stability of the released protein suggested that the preparation process of the EPLGA MPs did not affect the antigenicity and structure of the encapsulated RVLPs and LTB. Oral immunization results showed that RVLPs + LTB/EPLGA MPs was able to stimulate high levels of antigen-specific serum IgG, IgG1, IgG2a antibodies, serum cytokines IFN-γ and IL-4, and mucosal sIgA antibodies. Therefore, we may provide a promising strategy for the prevention of rabies in domestic animals by oral routes. What's more, this study also provides a platform for the oral delivery of other types of vaccines.

## Materials and methods

### Materials and animals

Poly-D, L-lactide-co-glycolide (PLGA, RESOMER®RG 503 H, 50:50, Mw 24–38 kDa) were obtained from Jinan Daigang Biomaterial Co., Ltd (Shandong, China). Poly-(vinyl alcohol) (PVA), Eudragit®L100, and 3-(4, 5-dimethylthiazol-2-yl)-2, 5-diphenyltetrazolium bromide (MTT) were supplied by Aladdin (Shanghai, China). Human embryonic kidney (HEK-293) cells were acquired from the Type Culture Collection Committee of Chinese Academy of Sciences (Shanghai, China). Fetal bovine serum (FBS), Dulbecco's Modified Eagle Medium (DMEM), and penicillin–streptomycin (10,000 U/mL penicillin and 10 mg/mL streptomycin) were purchased from Gibco Life Technologies (Grand Island, USA). Lipofectamine 3000 was bought from Thermo Fisher Scientific (Massachusetts, USA). Hoechst 33,342 was a product of the Beyotime Institute of Biotechnology (Shanghai, China). Rabies virus glycoprotein and matrix protein antibody were purchased from Epigentek (New York, USA). HRP conjugated goat anti-Mouse IgG/IgG1/IgG2a/sIgA antibody were obtained from Abcam (Cambridge, US). Commercial vaccine Rabisin was supplied by Merial (Georgia, USA). Mouse IL-4/IFN-γ Elisa kit was supplied by eBioscience (California, USA). APC anti-mouse CD8α antibody and FITC anti-mouse CD4 antibody were products of Biolegend (Shanghai, China). All other chemicals used in the study were of analytical grade.

### Analysis of candidate antigen rabies virus glycoprotein (RVGP)

The protein sequence of rabies virus glycoprotein (RVGP) (Accession: AAP81751.1) and matrix protein (RVMP) (Accession: ACA03545.1) of the challenge virus strain (CVS) were obtained on NCBI (https://www.ncbi.nlm.nih.gov/). To demonstrate whether RVGP is the optimal one as a broad-spectrum vaccine candidate against Chinese rabies, its protein sequence was compared with another 34 RVGP (NCBI) from different regions of China through DNAMAN software. Then, online software was used to predict the epitopes of RVGP, which including ABCpred prediction server (http://crdd.osdd.net/raghava/abcpred/), BioXGEM PAComplex (http://pacomplex.life.nctu.edu.tw./), and SYFPEITHI (http://www.syfpeithi.de/bin/MHCServer.dll/FindYourMotif.htm). Besides, the potential glycosylation sites of candidate RVGP were predicted by NetNGlyc 1.0 server (http://www.cbs.dtu.dk/services/NetNGlyc/).

### Design and construction of recombinant plasmids

RVMP, RVGP, and EGFP were cloned into the eukaryotic expression vector pcDNA3.1(+), named pcDNA3.1-RVLPs-EGFP. To improve its self-assembly efficiency and to avoid the unfavourable potential effect of fusion expression on antigenicity, each gene was designed with an independent promoter (CMV), ribosome binding site (GCCACC), and poly-A tail (PA). EGFP was used to monitor the stable expression of RVLPs. Besides, to further boost the immunogenicity of the RVLPs, the mucosal adjuvant E. coli heat-resistant enterotoxin B subunit (LTB) was expressed in E. coli and purified using nickel columns.

### Cell culture and transfection

HEK-293 cells were cultured in DMEM medium (Gibco) supplemented with 10% fetal bovine serum, 100 U/mL penicillin (Gibco), and 100 µg/mL streptomycin (Gibco) at 37°C with 5% CO2. Cells at a density of 2 × 10^6^ cells/well were trypsinized and seeded into 24-well plates for 24 h. Briefly, a mixture of 500 ng pcDNA3.1(+)-RVLPs-EGFP plasmids and 0.75 µL Lipofectamine 3000 was added into each well.

### Quantitative real-time PCR (qPCR) assay

Total RNA was extracted from HEK-293 cells transiently transfected with pcDNA3.1(+)-RVLPs-EGFP using the RNAiso Plus kit (Takara, Japan). Subsequently, 500 ng of extracted RNA was reverse transcribed into cDNA using a PrimeScript RT Reagent Kit with the genomic DNA Eraser (Takara, Japan). qPCR reactions were performed on the Bio-Rad CFX96 qPCR (BioRad, USA) using SYBR Premix Ex Taq. The programme for qPCR amplification was as follows: 95°C for 5 s, followed by 40 cycles at 95°C for 10 s, 55°C for 10 s, and 72°C for 20 s. The primers used are listed in Table S2.

### Western blotting (WB) assay

HEK-293 cells expressing RVLPs were lysed 48 h after transfection, and the culture medium was collected and concentrated using a 30 kDa ultrafiltration tube (Millipore, USA). The samples of cell lysis supernatant and concentrated culture medium were electrophoresed by SDS-PAGE (12%). After electro-transfer of the gel to a PVDF membrane, the membrane was blocked with 3% nonfat milk for 30 min at room temperature. The membrane was then incubated with anti-RVGP and anti-RVMP antibodies at 4°C overnight, followed by incubation with the secondary antibody for 1 h at room temperature. After each step, the PVDF membrane was washed with TBST for three times. Protein bands were visualized with the ultrasensitive ECL chemiluminescence kit (Sangon Biotech, China).

### MTT assay

HEK-293 cells (1 × 10^6^ cells/well) were seeded into 96-well plates and cultured overnight. The culture supernatant was removed and replaced with a fresh DMEM supplemented with different concentrations of G418. After 48 h of incubation, 20 µL MTT solution (5 mg/mL) was added to each well to determine the cell viability. Absorbance was detected at 490 nm.

### Stable cell line generation

Positive monoclonal cell lines were obtained by the limited dilution method. Briefly, 48 h after HEK-293 cells were transfected with pcDNA3.1(+)-RVLPs-EGFP plasmid, the cells were incubated with 2 mg/mL G418 in DMEM for 2 weeks. Cell clusters with green fluorescence were then digested and diluted to 1 cell/well before being seeded into 96-well plates. DMEM containing 1 mg/mL G418 was added into 96-well plates 48 h later. For approximately 1 week, the monoclonal wells were screened and identified by western blot and flow cytometry (FCM) (Becton Dickinson, USA). The stable cell line was named HEK-293/RVLPs. In addition, the stability of HEK-293/RVLPs cells was evaluated by western blot, FCM, and confocal laser scanning microscope (CLSM) (Hitachi, Japan) at the tenth generation (P10).

For flow cytometry (FCM) analysis, the HEK-293/RVLPs cells were digested by trypsin, then washed 3 times with 1 mL PBS and analyzed at the FITC channel (excitation: 488 nm and emission: 525 nm).

For confocal laser scanning microscopy (CLSM) analysis, the HEK-293/RVLPs cells were plated and fixed with 4% (v/v) paraformaldehyde in PBS for 10 min after adherence. After washing three times with PBS, the cells were incubated with Hoechst 33,342 for 10 min. Subsequently, the cells were washed 3 times with PBS and imaged by CLSM at FITC (excitation: 488 nm and emission: 525 nm) and Hoechst 33,342 channels (excitation: 350 nm and emission: 461 nm).

### Purification and characterization of RVLPs

HEK-293/RVLPs cells were lysed using a lysis buffer. The culture medium was filtered with 0.45 µm filter membrane and concentrated using a 30 kDa ultrafiltration tube. The RVLPs were purified by sucrose density gradient (20–40–60%) centrifugation (4°C, 15,000 × g, 3 h). After centrifugation, the white band between the 40–60% sucrose layer was collected and dissolved in PBS for centrifugation (4°C, 11,000 × g, 3 h) again. Finally, the concentration of the purified RVLPs in PBS was measured using a BCA assay kit (Sangon, China).

The morphology of the RVLPs was observed by transmission electron microscope (TEM) JEM-1400 (Jeol, Japan). Briefly, the purified RVLPs were adsorbed on a formvar-coated 300-mesh copper grid for 15–20 min. The grids were then negatively stained with 1% uranyl acetate for 30 s before examination by TEM (120 kV).

The cellular localization of RVLPs was also observed by TEM (200 kV). Briefly, mung beans sized HEK-293/RVLPs cells were collected and fixed with pre-cooled 2.5% electron microscope fixative solution overnight at 4°C. Subsequently, the samples were dehydrated through graded ethanol solutions and embedded in epoxy resin for the preparation of ultrathin cell sections, followed by staining with uranyl acetate and lead citrate.

### Preparation of RVLPs/EPLGA MPs

RVLP/EPLGA MPs were prepared as described previously with moderate modification (Graphical Abstract). Briefly, RVLPs (5 mg/mL, 200 µL) were added into the organic phases containing 25 mg Eudragit®L100 and 50 mg PLGA. The mixture was then vortexed for 3 min to generate a water-in-oil (O/W) emulsion. The O/W emulsion was then added dropwise to 1% PVA solution (20 mL) under magnetic stirring to form a water-in-oil-in-water (W/O/W) emulsion. The W/O/W emulsion was magnetically stirred overnight at 4°C to prepare the RVLP/EPLGA MPs.

### In vitro characterization of RVLPs/EPLGA MPs

The morphology of RVLPs/EPLGA MPs was observed by scanning electron microscope (SEM) (Hitachi, Japan). The encapsulation efficiency (EE) of RVLPs/EPLGA MPs was calculated by determining the residual concentration of RVLPs in PBS and using the following equations:

$$\mathrm{EE}\text{\%}=\frac{\mathrm{Initial}\;\mathrm{amount}\;\mathrm{of}\;\mathrm{RVLPs}\;\mathrm{added}-\mathrm{Amount}\;\mathrm{of}\;\mathrm{RVLPs}\;\mathrm{in}\;\mathrm{supernatant}}{\mathrm{Initial}\;\mathrm{amount}\;\mathrm{of}\;\mathrm{RVLPs}\;\mathrm{added}}\times 100\text{\%}$$

The stability of RVLPs and LTB encapsulated in the EPLGA MPs was determined by fourier transform infrared spectrometer (FTIR) (Nicolet 6700, Thermo Nicolet Corporation, USA) and circular dichroism (CD) (Chirascan, Applied Photophysics Ltd, UK).

### In vitro release and storage stability studies of RVLPS/EPLGA MPs

In vitro release profile of RVLPs/EPLGA MPs was evaluated in simulated gastric fluid (PBS, pH 1.2) and intestinal fluid (PBS, pH 6.8). Briefly, 10 mg of the freeze-dried RVLPs/EPLGA MPs were dissolved in 1 mL PBS and placed in an incubator shaker (100 rpm, 37°C). The supernatant was collected at predetermined time points (0.5, 1, and 2 h in pH 1.2 PBS, followed by 4, 6, 8, 12, and 24 h in pH 6.8 PBS). The concentration of free RVLPs in the supernatant was determined using the BCA assay kit. Besides, the RVLPs in the concentrated supernatant were also observed by TEM (120KV). In addition, the freeze-dried RVLPs/EPLGA MPs were sealed and stored at 4, 25, and 37°C for 3 months for stability comparison.

### The intestinal retention time of mCherry/EPLGA MPs

To evaluate the intestinal targeting of EPLGA MPs, mCherry/EPLGA MPs were prepared. BALB/c mice were fasted for 2 h with normal water supply, and then 100 µL of the mCherry/EPLGA MPs solution was administrated intragastrically. The mice were euthanized separately at 0.5, 1.5, 3, 5, and 10 h, and the gastrointestinal organs were dissected and observed using an *in vivo* imaging system (IVIS) (KODAK, USA). The excitation and emission wavelengths of the mCherry protein were 580 and 610 nm, respectively.

### Immunological studies

Female BALB/c mice (6–8 weeks of age) were purchased from Shanghai SLAC Laboratory Animal Co., Ltd (Shanghai, China). All experiments involving animals were approved by Chinese legislation on the use and care of research animals (Document No. 55, 2001) and controlled by the animal ethics committee of East China University of Science and Technology (REC No. 20181223). The mice were randomly divided into 10 groups (*n* = 5). They were allowed free access to food and water with a 12 h light/dark cycle. Before the immunization, mice were fasted but supplied water for 2 h and then immunized with various formulations by intragastric administration (Table S1), followed by a booster doses every 2 weeks for a total of three administrations.

### Collection of serum and intestinal

Blood from the retro-orbital plexus and faecal samples of mice were collected before and every two weeks after immunization. Serum was obtained by centrifugation of the blood samples and stored at −80°C for the detection of IgG, IgG1, and IgG2a antibodies (Abcam) and IL-4 and IFN-γ cytokines (eBioscience). Peripheral blood samples collected after the third immunization were used for the detection of CD4+ and CD8+ T cells. Moreover, major organs (heart, liver, spleen, lung, kidney, and intestine) were harvested and fixed with 4% paraformaldehyde for paraffin slicing, followed by haematoxylin-eosin (HE) staining for histological examination. Additionally, feces and intestine were dissolved with PBS and the supernatant of each was collected to test the RVLPs-specific sIgA antibodies (Biolegend).

### Enzyme-linked immunosorbent assay (ELISA)

The RVLPs-specific antibodies were detected by ELISA. Briefly, 96-well plates were coated with 100 µL of RVLPs (5 µg/mL) overnight at 4°C. Subsequently, the plates were blocked with 200 µL of blocking buffer (1% BSA in PBST) at 37°C for 2 h. Serum, intestinal, and fecal supernatants (100 µL) were added into the palates for 1.5 h at 37°C. HRP-conjugated goat anti-mouse IgG, IgG1, IgG2a, and sIgA antibodies (100 µL) were added to each well for another 2 h at 37°C. Thereafter, 100 µL of tetramethylbenzidine (TMB) was added, and 1 M H2SO4 (50 µL) was used to stop the reaction after 20 min. The absorbance was measured at OD450 nm. The plates were also washed six times with PBST (0.05% Tween 20 in PBS) after each step. Cytokines were measured according to the instructions of the IL-4 and IFN-γ kits (eBioscience).

### Statistical analyzes

All data were statistically analyzed using SPSS 22.0 software, flow charts were plotted by ChemBioOffice and data were processed using Origin 8.0 and GraphPad Prism 8 software. All experiments were performed at least three times. Data were expressed as mean ± SD. The student's *t*-test was used to compare the two groups, while one-way or two-way ANOVA was used for multi-group comparisons to determine statistically significant differences between the samples. The *P* value < 0.05 was significant, and the significance level was shown in the figures as *P < 0.05, **P < 0.01, ***P < 0.001.

## Supplementary Material

Graphical abstract.jpg

Supplementary materials.pdf

## Data Availability

Data supporting the conclusions of this study can be found in this article or the Supplementary information. Any additional data are available from the corresponding author upon reasonable request.

## References

[1] Minghui R, Stone M, Semedo MH, et al. New global strategic plan to eliminate dog-mediated rabies by 2030. Lancet Glob Health. 2018;6(8):e828–e829. doi:10.1016/S2214-109X(18)30302-4 29929890

[2] Wallace RM, Cliquet F, Fehlner-Gardiner C, et al. Role of oral rabies vaccines in the elimination of dog-mediated human rabies deaths. Emerg Infect Dis. 2020;26(12):1–9. doi:10.3201/eid2612.201266 PMC7706920 33219786

[3] Fisher CR, Streicker DG, Schnell MJ. The spread and evolution of rabies virus: conquering new frontiers. Nat Rev Microbiol. 2018;16(4):241–255. doi:10.1038/nrmicro.2018.11 29479072 PMC6899062

[4] Fooks AR, Cliquet F, Finke S, et al. Rabies. Nat Rev Dis Primers. 2017;3:17091. doi:10.1038/nrdp.2017.91 29188797

[5] Cauchemez S, Bourhy H. Improving the provision of rabies post-exposure prophylaxis. Lancet Infect Dis. 2019;19(1):12–13. doi:10.1016/S1473-3099(18)30606-6 30472177

[6] Schnell MJ, McGettigan JP, Wirblich C, et al. The cell biology of rabies virus: using stealth to reach the brain. Nat Rev Microbiol. 2010;8(1):51–61. doi:10.1038/nrmicro2260 19946287

[7] Davis BM, Rall GF, Schnell MJ. Everything you always wanted to know about rabies virus (but were afraid to ask). Annu Rev Virol. 2015;2(1):451–471. doi:10.1146/annurev-virology-100114-055157 26958924 PMC6842493

[8] Maki J, Guiot AL, Aubert M, et al. Oral vaccination of wildlife using a vaccinia-rabies-glycoprotein recombinant virus vaccine (RABORAL V-RG(®)): a global review. Vet Res. 2017;48(1):57. doi:10.1186/s13567-017-0459-9 28938920 PMC5610451

[9] Fry TL, Vandalen KK, Duncan C, et al. The safety of ONRAB® in select non-target wildlife. Vaccine. 2013;31(37):3839–3842. doi:10.1016/j.vaccine.2013.06.069 23831321

[10] Kellogg F, Niehus N, DiOrio M, et al. Human contacts with oral rabies vaccine baits distributed for wildlife rabies management--Ohio, 2012. MMWR Morb Mortal Wkly Rep. 2013;62(14):267–269. 23575240 PMC4604900

[11] Vela Ramirez JE, Sharpe LA, Peppas NA. Current state and challenges in developing oral vaccines. Adv Drug Delivery Rev. 2017;114:116–131. doi:10.1016/j.addr.2017.04.008 PMC6132247 28438674

[12] Chen XY, Butt AM, Mohd Amin MCI. Enhanced paracellular delivery of vaccine by hydrogel microparticles-mediated reversible tight junction opening for effective oral immunization. J Controlled Release. 2019;311-312:50–64. doi:10.1016/j.jconrel.2019.08.031 31465827

[13] Brun A, Bárcena J, Blanco E, et al. Current strategies for subunit and genetic viral veterinary vaccine development. Virus Res. 2011;157(1):1–12. doi:10.1016/j.virusres.2011.02.006 21316403

[14] Ludwig C, Wagner R. Virus-like particles-universal molecular toolboxes. Curr Opin Biotechnol. 2007;18(6):537–545. doi:10.1016/j.copbio.2007.10.013 18083549 PMC7126091

[15] Schwarz B, Douglas T. Development of virus-like particles for diagnostic and prophylactic biomedical applications. WIRES Nanomed Nanobiotechnol. 2015;7(5):722–735. doi:10.1002/wnan.1336 PMC4515192 25677105

[16] Mohsen MO, Zha L, Cabral-Miranda G, et al. Major findings and recent advances in virus-like particle (VLP)-based vaccines. Semin Immunol. 2017;34:123–132. doi:10.1016/j.smim.2017.08.014 28887001

[17] Balke I, Zeltins A. Use of plant viruses and virus-like particles for the creation of novel vaccines. Adv Drug Delivery Rev. 2019;145:119–129. doi:10.1016/j.addr.2018.08.007 30172923

[18] Fontana D, Marsili F, Garay E, et al. A simplified roller bottle platform for the production of a new generation VLPs rabies vaccine for veterinary applications. Comp Immunol Microbiol Infect Dis. 2019;65:70–75. doi:10.1016/j.cimid.2019.04.009 31300130

[19] De Benedictis P, Minola A, Rota Nodari E, et al. Development of broad-spectrum human monoclonal antibodies for rabies post-exposure prophylaxis. EMBO Mol Med. 2016;8(4):407–421. doi:10.15252/emmm.201505986 26992832 PMC4818751

[20] Qi Y, Kang H, Zheng X, et al. Incorporation of membrane-anchored flagellin or Escherichia coli heat-labile enterotoxin B subunit enhances the immunogenicity of rabies virus-like particles in mice and dogs. Front Microbiol. 2015;6:169. 25784906 10.3389/fmicb.2015.00169 PMC4347500

[21] Kang H, Qi Y, Wang H, et al. Chimeric rabies virus-like particles containing membrane-anchored GM-CSF enhances the immune response against rabies virus. Viruses. 2015;7(3):1134–1152. doi:10.3390/v7031134 25768031 PMC4379564

[22] Tom JK, Albin TJ, Manna S, et al. Applications of immunomodulatory immune synergies to adjuvant discovery and vaccine development. Trends Biotechnol. 2019;37(4):373–388. doi:10.1016/j.tibtech.2018.10.004 30470547

[23] Schijns V, Fernández-Tejada A, Barjaktarović Ž, et al. Modulation of immune responses using adjuvants to facilitate therapeutic vaccination. Immunol Rev. 2020;296(1):169–190. doi:10.1111/imr.12889 32594569 PMC7497245

[24] Coffey JW, Gaiha GD, Traverso G. Oral biologic delivery: advances toward oral subunit, DNA, and mRNA vaccines and the potential for mass vaccination during pandemics. Annu Rev Pharmacol Toxicol. 2021;61:517–540. doi:10.1146/annurev-pharmtox-030320-092348 32466690 PMC8057107

[25] Patry RT, Stahl M, Perez-Munoz ME, et al. Bacterial AB(5) toxins inhibit the growth of gut bacteria by targeting ganglioside-like glycoconjugates. Nat Commun. 2019;10(1):1390. doi:10.1038/s41467-019-09362-z 30918252 PMC6437147

[26] Jain RA. The manufacturing techniques of various drug loaded biodegradable poly(lactide-co-glycolide) (PLGA) devices. Biomaterials. 2000;21(23):2475–2490. doi:10.1016/S0142-9612(00)00115-0 11055295

[27] Akagi T, Baba M, Akashi M. Biodegradable nanoparticles as vaccine adjuvants and delivery systems: regulation of immune responses by nanoparticle-based vaccine. Adv Polym Sci. 2011;247(1):31–64. doi:10.1007/12_2011_150

[28] Fu K, Pack DW, Klibanov AM, et al. Visual evidence of acidic environment within degrading poly(lactic-co-glycolic acid) (PLGA) microspheres. Pharm Res. 2000;17(1):100–106. doi:10.1023/A:1007582911958 10714616

[29] Rajasree PH, Paul W, Sharma CP, et al. Eudragit encapsulated cationic poly(lactic- co -glycolic acid) nanoparticles in targeted delivery of capecitabine for augmented colon carcinoma therapy. J Drug Deliv Sci Technol. 2018;46:302–311. doi:10.1016/j.jddst.2018.05.025

[30] Hu F, Yan T, Guo W, et al. Multiple targeting strategies achieve novel protein drug delivery into proapoptosis lung cancer cells by precisely inhibiting survivin. Nanoscale. 2020;12(19):10623–10638. doi:10.1039/D0NR01352H 32373859

[31] Fernandes C, Martins C, Fonseca A, et al. PEGylated PLGA nanoparticles as a smart carrier to increase the cellular uptake of a coumarin-based monoamine oxidase B inhibitor. ACS Appl Mater Interfaces. 2018;10(46):39557–39569. doi:10.1021/acsami.8b17224 30352150

[32] Jain NK, Sahni N, Kumru OS, et al. Formulation and stabilization of recombinant protein based virus-like particle vaccines. Adv Drug Delivery Rev. 2015;93:42–55. doi:10.1016/j.addr.2014.10.023 25451136

[33] Roy K, Kanwar RK, Krishnakumar S, et al. Competitive inhibition of survivin using a cell-permeable recombinant protein induces cancer-specific apoptosis in colon cancer model. Int J Nanomed. 2015;10:1019–1043. doi:10.2217/nnm.14.201 PMC4324544 25678789

[34] Fan K, Jiang B, Guan Z, et al. Fenobody: a ferritin-displayed nanobody with high apparent affinity and half-life extension. Anal Chem. 2018;90(9):5671–5677. doi:10.1021/acs.analchem.7b05217 29634235

[35] Wang S, Liu H, Zhang X, et al. Intranasal and oral vaccination with protein-based antigens: advantages, challenges and formulation strategies. Protein Cell. 2015;6(7):480–503. doi:10.1007/s13238-015-0164-2 25944045 PMC4491048

[36] Wahlin BE, Sander B, Christensson B, et al. Entourage: the immune microenvironment following follicular lymphoma. Blood Cancer J. 2012;2(1):e52. doi:10.1038/bcj.2011.53 22829236 PMC3270257

[37] Cantaert T, Borand L, Kergoat L, et al. A 1-week intradermal dose-sparing regimen for rabies post-exposure prophylaxis (RESIST-2): an observational cohort study. Lancet Infect Dis. 2019;19(12):1355–1362. doi:10.1016/S1473-3099(19)30311-1 31570311

[38] de Melo GD, Sonthonnax F, Lepousez G, et al. A combination of two human monoclonal antibodies cures symptomatic rabies. EMBO Mol Med. 2020;12(11):e12628. doi:10.15252/emmm.202012628 32945125 PMC7645379

[39] Zhang W, Zheng X, Cheng N, et al. Isatis indigotica root polysaccharides as adjuvants for an inactivated rabies virus vaccine. Int J Biol Macromol. 2016;87:7–15. doi:10.1016/j.ijbiomac.2016.02.023 26875535 PMC7112441

[40] Undurraga EA, Blanton JD, Thumbi SM, et al. Tool for eliminating dog-mediated human rabies through mass dog vaccination campaigns. Emerg Infect Dis. 2017;23(12):2114–2116. doi:10.3201/eid2312.171148 29148385 PMC5708230

[41] Yang F, Lin S, Ye F, et al. Structural analysis of rabies virus glycoprotein reveals pH-dependent conformational changes and interactions with a neutralizing antibody. Cell Host Microbe. 2020;27(3):441–453.e7. doi:10.1016/j.chom.2019.12.012 32004500

[42] Astray RM, Jorge SA, Pereira CA. Rabies vaccine development by expression of recombinant viral glycoprotein. Arch Virol. 2017;162(2):323–332. doi:10.1007/s00705-016-3128-9 27796547

[43] Lalonde ME, Durocher Y. Therapeutic glycoprotein production in mammalian cells. J Biotechnol. 2017;251:128–140. doi:10.1016/j.jbiotec.2017.04.028 28465209

[44] Zhang L, Lua LH, Middelberg AP, et al. Biomolecular engineering of virus-like particles aided by computational chemistry methods. Chem Soc Rev. 2015;44(23):8608–8618. doi:10.1039/C5CS00526D 26383145

[45] Fontana D, Kratje R, Etcheverrigaray M, et al. Rabies virus-like particles expressed in HEK293 cells. Vaccine. 2014;32(24):2799–2804. doi:10.1016/j.vaccine.2014.02.031 24631077

[46] Fernández-Núñez EG, de Rezende AG, Puglia AL, et al. Transient expression of rabies virus G-glycoprotein using BHK-21 cells cultured in suspension. Biotechnol Lett. 2015;37(6):1153–1163. doi:10.1007/s10529-015-1787-3 25700821

[47] Kour P, Rath G, Sharma G, et al. Recent advancement in nanocarriers for oral vaccination. Artif Cells Nanomed Biotechnol. 2018;46(sup3):S1102–s1114. doi:10.1080/21691401.2018.1533842 30453779

[48] Patil YP, Jadhav S. Novel methods for liposome preparation. Chem Phys Lipids. 2014;177:8–18. doi:10.1016/j.chemphyslip.2013.10.011 24220497

[49] Maghrebi S, Prestidge CA, Joyce P. An update on polymer-lipid hybrid systems for improving oral drug delivery. Expert Opin Drug Delivery. 2019;16(5):507–524. doi:10.1080/17425247.2019.1605353 30957577

[50] Kole S, Qadiri SSN, Shin SM, et al. PLGA encapsulated inactivated-viral vaccine: formulation and evaluation of its protective efficacy against viral haemorrhagic septicaemia virus (VHSV) infection in olive flounder (Paralichthys olivaceus) vaccinated by mucosal delivery routes. Vaccine. 2019;37(7):973–983. doi:10.1016/j.vaccine.2018.12.063 30661835

[51] Lim M, Badruddoza AZM, Firdous J, et al. Engineered nanodelivery systems to improve DNA vaccine technologies. Pharmaceutics. 2020;12(1):30. doi:10.3390/pharmaceutics12010030 31906277 PMC7022884

[52] Zhu Q, Talton J, Zhang G, et al. Large intestine-targeted, nanoparticle-releasing oral vaccine to control genitorectal viral infection. Nat Med. 2012;18(8):1291–1296. doi:10.1038/nm.2866 22797811 PMC3475749

[53] Waheed MT, Thönes N, Müller M, et al. Plastid expression of a double-pentameric vaccine candidate containing human papillomavirus-16 L1 antigen fused with LTB as adjuvant: transplastomic plants show pleiotropic phenotypes. Plant Biotechnol J. 2011;9(6):651–660. doi:10.1111/j.1467-7652.2011.00612.x 21447051

