# Supplementary figures and images for: Development of an oral gut-targeted rabies virus-like particles (RVLPs) vaccine with mucosal immune adjuvant LTB via delivering of localized-release microparticles
Source: Emerg Microbes Infect. 2025 Jun 6;14(1):2515406. doi: 10.1080/22221751.2025.2515406 (PMC12172084; doi:10.1080/22221751.2025.2515406)

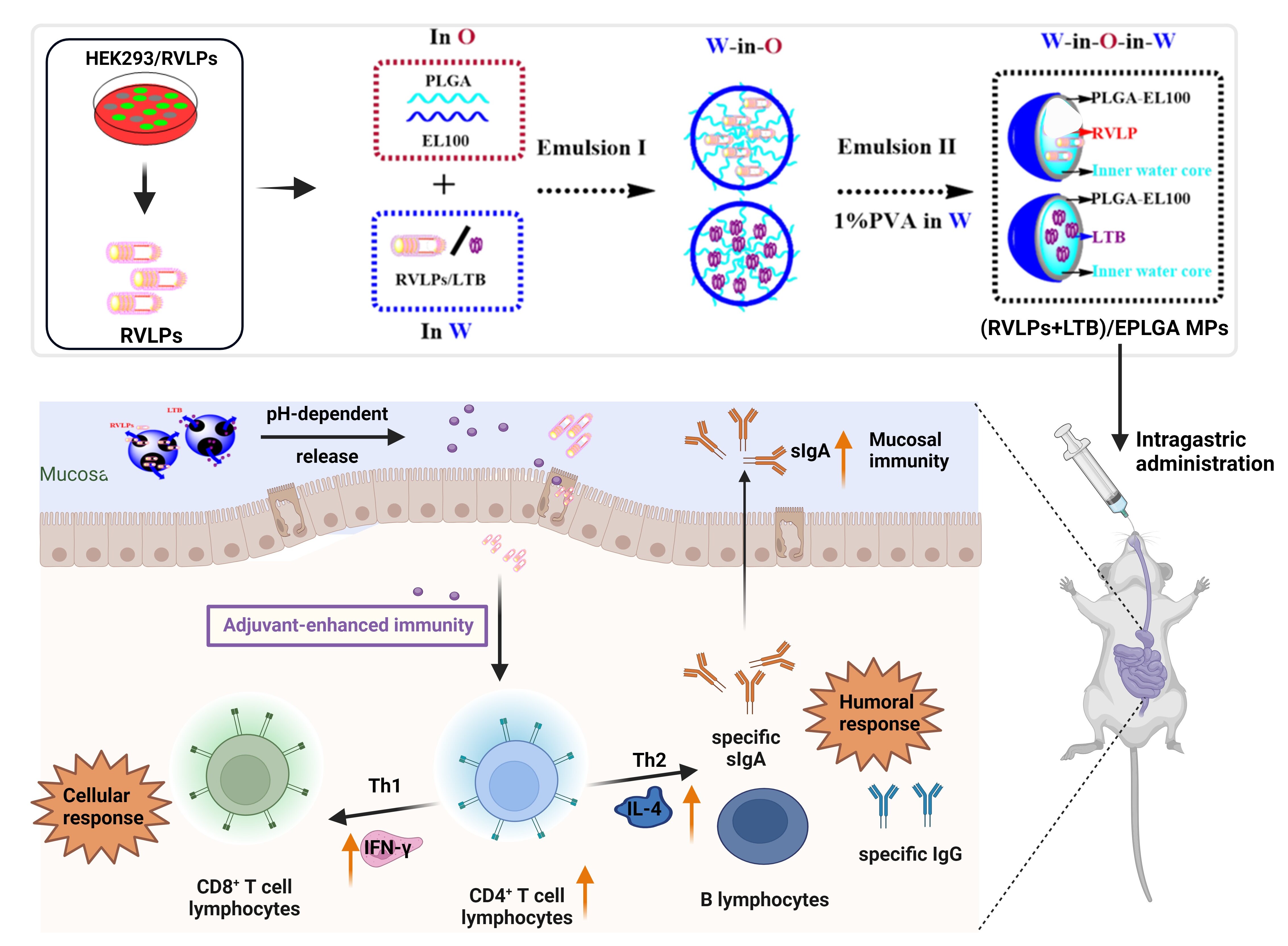

Supplement: Graphical abstract.jpg [file TEMI_A_2515406_SM5338.jpg]
