# Supplementary material for: Development of an oral gut-targeted rabies virus-like particles (RVLPs) vaccine with mucosal immune adjuvant LTB via delivering of localized-release microparticles
Source: Emerg Microbes Infect. 2025 Jun 6;14(1):2515406. doi: 10.1080/22221751.2025.2515406 (PMC12172084; doi:10.1080/22221751.2025.2515406)
Supplement: Supplementary materials.pdf [file TEMI_A_2515406_SM5337.pdf]

Supplementary Materials for

Development of an oral gut-targeted rabies virus-like particles (RVLPs) vaccine with mucosal immune adjuvant LTB via delivering of localized-release microparticles

Jinping Niu *et al.*

*Corresponding author Email: maxy@ecust.edu.cn

This PDF file includes:

Figs. S1 to S4
Tables S1 to S2

Figtures

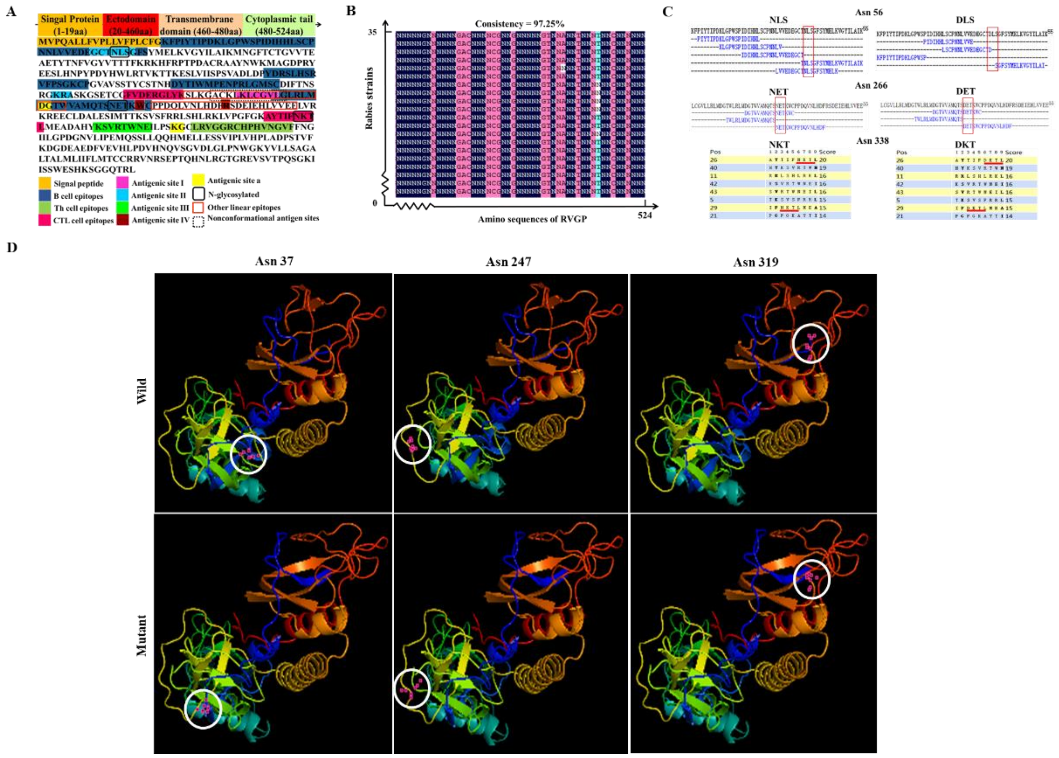

Fig S1. Rational analysis of candidate RVGP sequence from CVS strain. (A) The antigen epitopes and N-glycosylated sites of candidate RVGP. **(B)** Comparison of RVGP sequences between 34 Chinese rabies strains and candidate RVGP. The effects of glycosylation site mutation of RVGP on antigen epitopes **(C)** and its tertiary structure **(D)**.

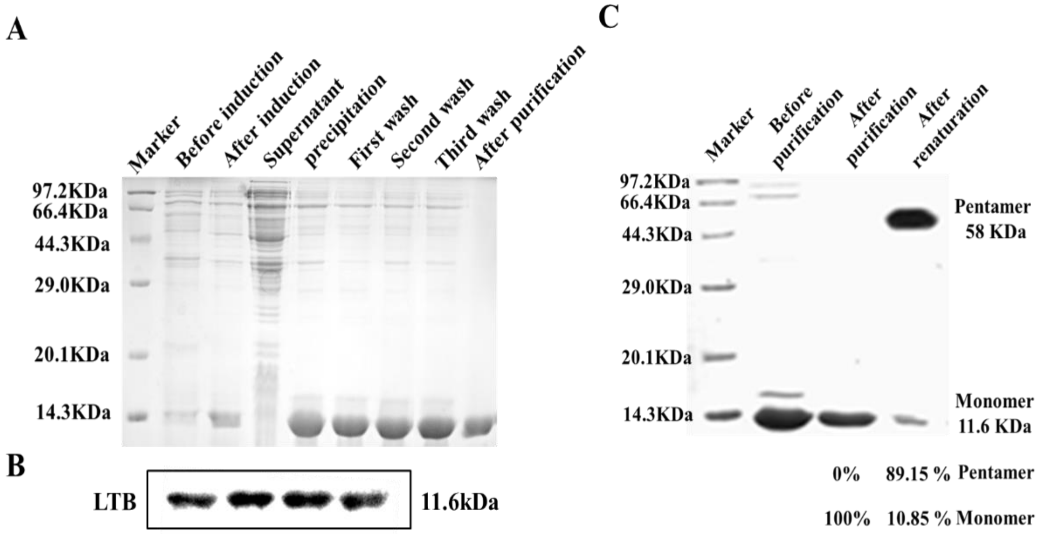

Fig S2. Expression and purification of recombinant protein LTB. Purification **(A)**, WB verification **(B)**, and renature **(C)** of LTB protein.

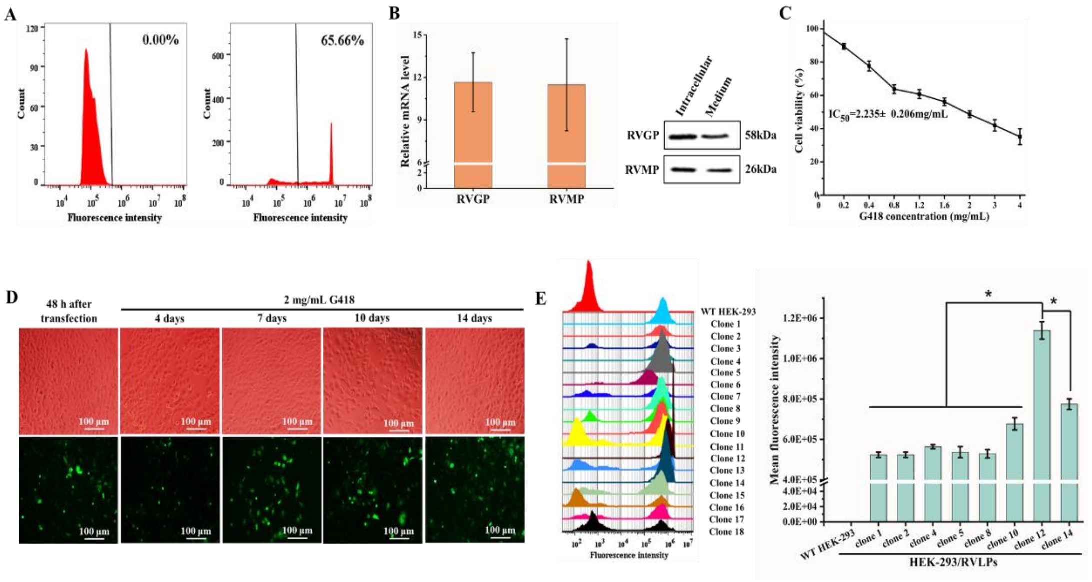

Fig S3. Screening and enrichment of positive cells. (A) The transfection efficiency of HEK-293 cells was detected by FCM. **(B)** Detection the RVLPs by qPCR and WB after 48 h transfection of HEK-293 cells. **(C)** Cell viability of HEK-293 cells incubated with different concentrations of G418 for 48 h. **(D)** The enrichment of positive cell lines. **(E)** The identification of positive cell lines. All images were taken at 100× magnification (Bar = 100 μm). Data were expressed as mean ± SD (n = 3).

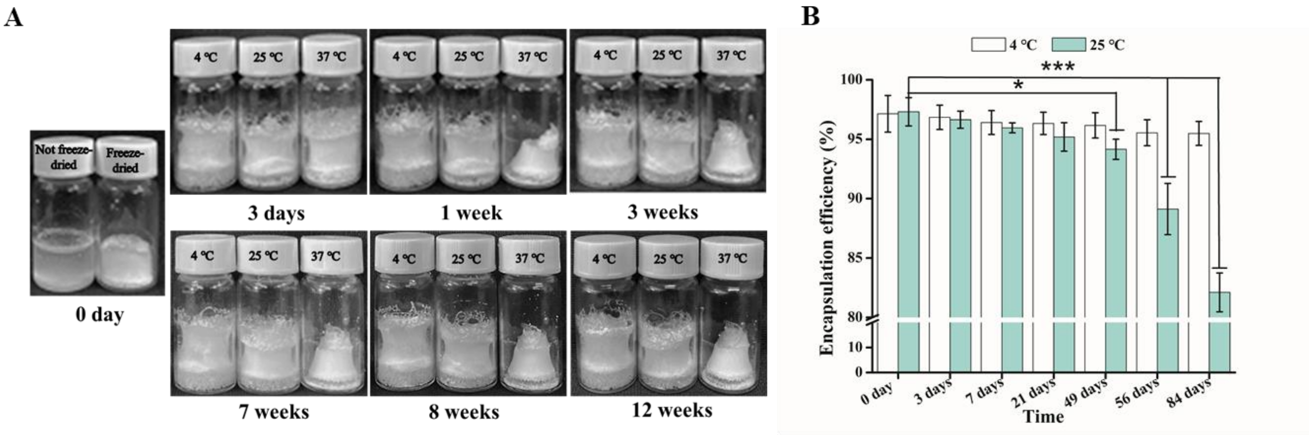

Fig S4. Storage stability analysis of RVLPs/EPLGA MPs. The appearance of RVLPs/EPLGA microspheres**(A)** and encapsulate efficiency **(B)** at different storage conditions.

Tables:

Table S1 Animal experimental group and immune dose.

Administration	Group	Dose	Number of mice
Intragastric (i.g.)	Saline	0 μg	5
	EPLGA MPs	100 μg	5
	LTB/ EPLGA MPs	50 μg	5
	RVLPs	100 μg	5
	RVLPs+LTB	100/50 μg	5
	RVLPs / EPLGA MPs	100 μg	5
	RVLPs+LTB / EPLGA MPs	100/50 μg	5
	(RVLPs+LTB / EPLGA	200/100 μg	5
	Rabisin (R)	200 μg	5
Intramuscular (i.m.)	Rabisin (R)	100 μg	5

Table S2. Primers list of qPCR

Gene	Primer sequence (5'-3')
RVGP	F: CTGCTCTACCAACCACGACTACAC
	R: GTCCACGAAGCCGCATGTCTC
RVMP	F: TGGATGACGATGACTTGTGGCTTC
	R: AACGAGTAACCATTCGGGCTACAC
β-actin	F: ATTGGCAATGAGCGGTTC
	R: ATACTCCTGCTTGCTGATCC
